# Exposure to anti-seizure medication during pregnancy and the risk of autism and ADHD in offspring: a systematic review and meta-analysis

**DOI:** 10.3389/fneur.2024.1440145

**Published:** 2024-07-22

**Authors:** Shan-Chun Xu, Ying Zhong, Hai-Yin Jiang, Jun Tang

**Affiliations:** ^1^Department of General Medicine, The First Affiliated Hospital of Zhejiang Chinese Medical University (Zhejiang Provincial Hospital of Traditional Chinese Medicine), Hangzhou, Zhejiang, China; ^2^Department of Orthopaedics, The First Affiliated Hospital of Zhejiang Chinese Medical University (Zhejiang Provincial Hospital of Traditional Chinese Medicine), Hangzhou, Zhejiang, China; ^3^State Key Laboratory for Diagnosis and Treatment of Infectious Diseases, Collaborative Innovation Center for Diagnosis and Treatment of Infectious Diseases, The First Affiliated Hospital, College of Medicine, Zhejiang University, Hangzhou, China

**Keywords:** mother, anticonvulsant, anti-epileptic, autistic, attention

## Abstract

**Background:**

Evidence of an association between maternal use of anti-seizure medication (ASM) during pregnancy and the risk of autism spectrum disorder (ASD) or attention-deficit/hyperactivity disorder (ADHD) in children is conflicting. This systematic review and meta-analysis aimed to summarize the relationship between fetal exposure to ASM and the development of ASD or ADHD in offspring.

**Methods:**

A comprehensive literature search was conducted in PubMed and other databases to identify relevant epidemiological studies published from inception until 1 March 2024.

**Results:**

Seven cohort studies were included in the meta-analysis. The results showed that maternal exposure to ASMs during pregnancy was associated with an increased risk of ASD [odds ratio (OR): 2.1, 95% confidence interval (CI): 1.63–2.71; *p* < 0.001] in the general population. This association became weaker (ASD: OR: 1.38, 95% CI: 1.11–1.73; *p* = 0.004) when the reference group was mothers with a psychiatric disorder or epilepsy not treated during pregnancy. Furthermore, an increased risk of ADHD was observed when the study data adjusted for drug indications were pooled (OR: 1.43, 95% CI: 1.07–1.92; *p* = 0.015). In subgroup analyses based on individual ASM use, only exposure to valproate preconception was significantly associated with an increased risk of ASD or ADHD.

**Conclusion:**

The significant association between maternal ASM use during pregnancy and ASD or ADHD in offspring may be partially explained by the drug indication or driven by valproate.

## Introduction

The use of anti-seizure medications (ASMs) has increased among pregnant mothers, with an almost three-fold increase seen in the worldwide during the past 12 years (1, 2). ASMs are used mainly for epilepsy during pregnancy; however, the greater number of prescriptions during pregnancy is driven largely by women with indications other than epilepsy, such as mood disorders or pain (1). Exposure to ASMs may lead to unwanted adverse maternal and neonatal outcomes (3); however, discontinuing the drug before or during pregnancy may lead to worsening or recurrence of the disease and thereby increase maternal mortality (4). Therefore, the decision to use an ASM during pregnancy is complex, and their reproductive safety should be understood.

ASMs theoretically pass through the human placenta and disrupt fetal neurodevelopment by changing the dopaminergic system (5). As dopamine receptors play a role in neurodevelopment (6), there are increasing concerns about adverse neurodevelopmental effects in offspring after prenatal exposure to ASMs. Animal studies have demonstrated that *in utero* exposure to these agents results in an increased risk of neurodevelopmental disorders, such as autism spectrum disorder (ASD) and attention-deficit/hyperactivity disorder (ADHD), in offspring (7). Accordingly, several epidemiological studies (8–14) have investigated the effect of ASMs on ASD or ADHD risk, but the results were inconsistent. In the earliest cohort study, Christensen et al. (8) observed an increased risk of ASD among offspring exposed to valproate but not among those exposed to other ASMs; however, ASD was reportedly associated with exposure to other ASMs during pregnancy in several other studies (10, 12, 13). Prenatal exposure to ASMs increases the risk of ADHD in offspring; however, the results are inconsistent.

Due to the increased use of ASMs during pregnancy, determining the long-term effects of maternal use of ASMs on the neurodevelopment of offspring, including possible effects on the incidence of ASD or ADHD, is important. To date, no systematic review or meta-analysis has been conducted on this topic, so we conducted the present study to assess the association between maternal ASM exposure during pregnancy and the subsequent risk of neurodevelopmental disorders in offspring.

## Methods

This systematic review and meta-analysis was conducted according to the Preferred Reporting Items for Systematic Review and Meta-Analyses (PRISMA) guidelines.

### Search strategy

A literature search was conducted of the PubMed and Embase databases on 1 March 2024, combining the following free-text words: (antiepileptic AND anti-seizure AND anticonvulsant) AND (autism OR autistic OR ASD OR attention-deficit/hyperactivity disorder OR ADHD OR attention-deficit disorder OR hyperkinetic disorder) AND (pregnancy OR mothers OR pregnant OR gestational OR prenatal OR perinatal OR gestation).

### Inclusion criteria

We analyzed peer-reviewed epidemiological studies that examined the relationship between maternal exposure to ASMs during pregnancy and the subsequent risk of ASD or ADHD in offspring. A study was eligible if it used a cohort or case–control design, included a non-ASM exposure reference group, reported either the relative risk or hazard ratio or odds ratio (OR), and contained data allowing us to estimate risk. We excluded case reports, case series, animal studies, correspondence, editorials, and reviews.

### Data extraction and quality assessment

Two reviewers independently extracted the data from the studies using an Excel spreadsheet, and any discrepancies were resolved by a third reviewer. The following data were collected from each study: first author, publication year, study location, study period, data source, information source for ASM exposure, exposure time, outcome measures, and statistical adjustments. Risk of bias and quality were assessed using the Newcastle-Ottawa scale (NOS), which was developed to assess the quality of epidemiological studies (15). This scale scores studies on three dimensions with nine questions relevant to research quality: the selection and comparability of the study groups and the relevance to the exposure (case–control) or outcome (cohort) of interest; all questions were scored as 0 or 1, and studies with scores ≥ 7 were considered to be of high quality.

### Outcome measurements

The primary outcome was the risk of ASD or ADHD with ASM use during pregnancy compared with no treatment in the general population. To rule out the potential effect of confounding by indication (bipolar disorder or epilepsy), the risk was assessed by comparing mothers with a clinical indication for taking an ASM to mothers with a clinical indication for not taking an ASM. To identify potential sources of heterogeneity, subgroup analyses were stratified according to the individual ASM.

### Statistical analysis

STATA 12.0 software (StataCorp LP, College Station, TX, United States) was utilized for all statistical analyses. Between-study heterogeneity was assessed using the χ^2^ test and I^2^ statistic; we considered I^2^ > 50% or *p* < 0.05 for the Q-statistic to indicate high heterogeneity (16). Pooled ORs with 95% confidence intervals (CIs) for dichotomous data were calculated using random or fixed-effects models according to the heterogeneity of the studies. We used a random effects model because its assumptions account for high heterogeneity among studies; otherwise, a fixed effects model was used (17). Publication bias was evaluated by Begg’s funnel plot (18). The funnel plot was not used to assess publication bias in analyses of <10 studies (19). We considered *p* < 0.05 to indicate a significant difference.

## Results

### Search results

**Figure 1:**
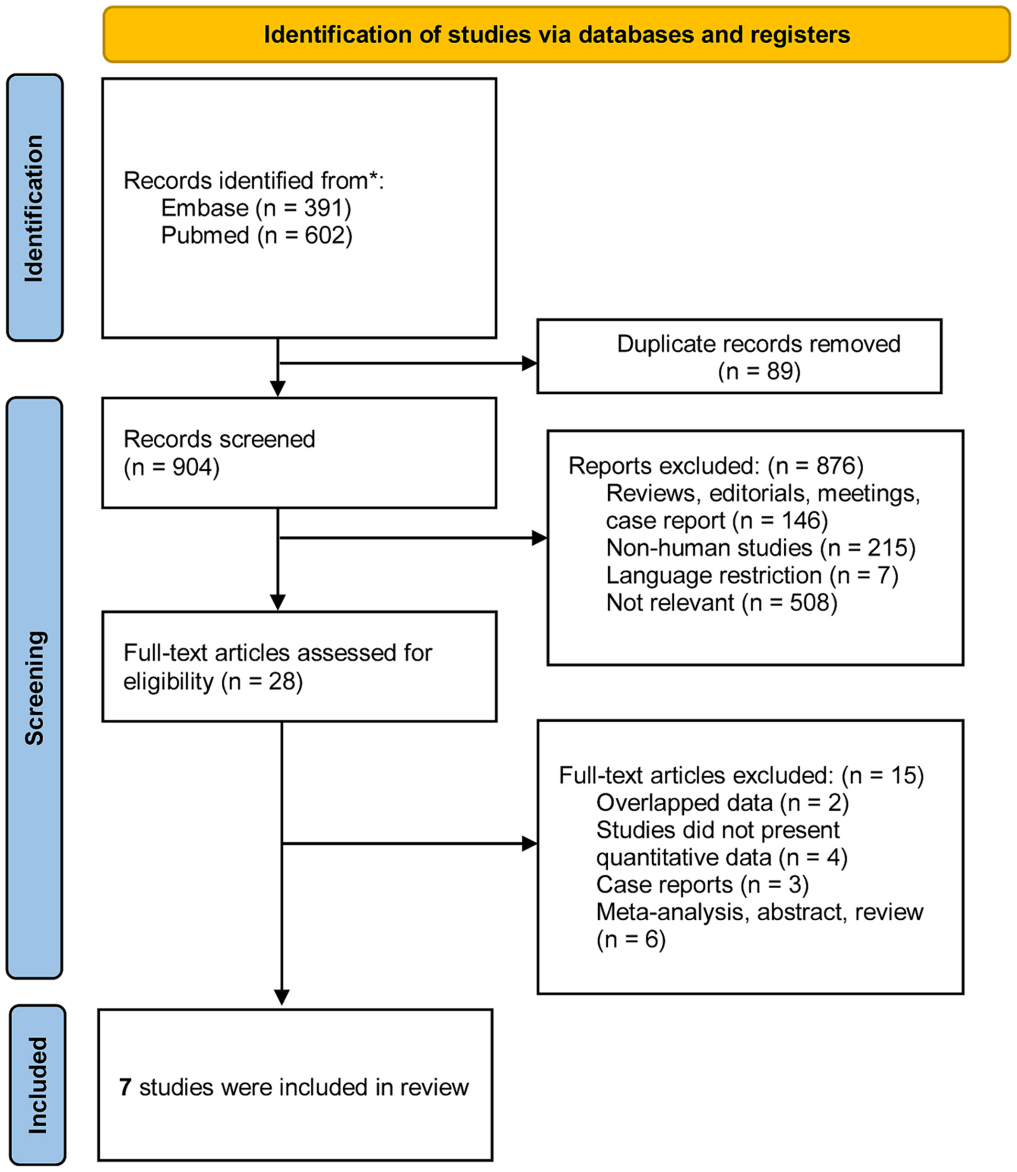
Flow-chart of the studies considered and finally selected for review.

The study selection process is shown in the PRISMA flow diagram (Figure 1). The initial search resulted in 993 citations, of which 89 were duplicates discarded before screening. A total of 876 articles were removed due to irrelevance to the research question, and 28 studies were included in the full-text review stage. Ultimately, seven publications fulfilled the eligibility criteria and were included in the present review.

### Characteristics of the included studies

Table 1 summarizes the nine studies considered in this review. The studies included 10,370,996 mothers from three different continents: five from Europe, one from North America, and one study from Asia. The sample size of the studies ranged from 38,661 to 4,494,926, and the publication year ranged from 2013 to 2024. Four studies evaluated these associations in mothers with epilepsy or the general population; two studies only assessed these associations in mothers with epilepsy; and the remaining study only evaluated these associations in mothers with bipolar disorder. There was heterogeneity in the type of ASM used among the studies; the ASMs included valproate, oxcarbazepine, lamotrigine, clonazepam, carbamazepine, oxcarbazepine, levetiracetam and topiramate. All studies scored > 7 on the NOS scale, indicating high overall quality (Supplementary Table S1).

**Table 1:** Characteristics of the included studies.

Author, year	Locating/setting	Study design/Born period of the children	Age	Ascertainment of ASMs exposure	Outcome reported	Outcome measurement	Source of diagnosis	Number of mothers	Confounding adjusted	Quality
Christensen et al. (8)	Denmark, population-based	Cohort study/1996–2006	<14	Danish Prescription Register	ASD	ICD-10	Danish Psychiatric Central Register	655,615	Maternal age at conception, paternal age at conception, parental psychiatric history, gestational age, birth weight, sex, congenital malformations, and parity	9
Christensen et al. (9)	Denmark, population-based	Cohort study/1997–2011	<18	Danish Prescription Register	ADHD	ICD-10 or use of ADHD medication	Danish Psychiatric Central Register or Danish Prescription Register	913,302	Maternal age at conception, maternal psychiatric history, maternal epilepsy, maternal diabetes, sex of the child, year of birth, and parity.	9
Wiggs et al. (10)	Sweden, population-based	Cohort study/1996–2013	<17	Prescribed Drug Register	ASD, ADHD	ICD-based diagnoses	National Patient Register	14,614	Year of birth, birth order, child sex, and maternal-reported smoking during pregnancy, maternal and paternal characteristic, parental psychiatric and behavioral problems	9
Yeh et al. (11)	Taiwan, population-based	Cohort study/1996–2013	<9	Taiwan National Health Insurance	ASD, ADHD	ICD-9-CM	Taiwan National Health Insurance	11,338	Demographic characteristics	7
Bjørk et al. (12)	Denmark, Finland, Iceland, Norway, and Sweden, population-based	Cohort study/1996–2017	<11	National prescription registers	ASD	ICD-10	Nordic health registers	4,494,926	Maternal age, education and marital status, parity, child’s birth year, sex, and country of birth, maternal use of antidepressants or opioids, depression, anxiety, personality disorders, number of chronic somatic diseases, and number of hospitalizations the year before last menstrual period	9
Dreier et al. (13)	Denmark, Finland, Iceland, Norway, and Sweden, population-based	Cohort study/1996–2017	<22	National prescription registers	ASD, ADHD	ICD-10	Nordic health registers	38,661	Maternal age, parity, educational level, smoking in pregnancy, use of antidepressants in pregnancy, and maternal psychiatric comorbidity	9
Hernández-Díaz et al. (14)	USA, population-based	Cohort study/2000–2018	<20	MAX-TAF or MarketScan	ASD	A validated claims-based algorithm that requires at least two visits with codes	Health registers	4,292,539	Demographic characteristics, maternal mental health and neurologic conditions other than epilepsy, other potential indications, concomitant medications, lifestyle factors, maternal coexisting conditions, and health care use	9

ADHD, attention deficit hyperactivity disorder; ASD, autism spectrum disorder; MAX-TAF, Medicaid Analytic eXtract–Transformed Medicaid Statistical Information System Analytic Files; ICD, International Classification of Diseases.

### Meta-analysis

#### Associations between ASM use and ASD and ADHD in the general population

**Figure 2:**
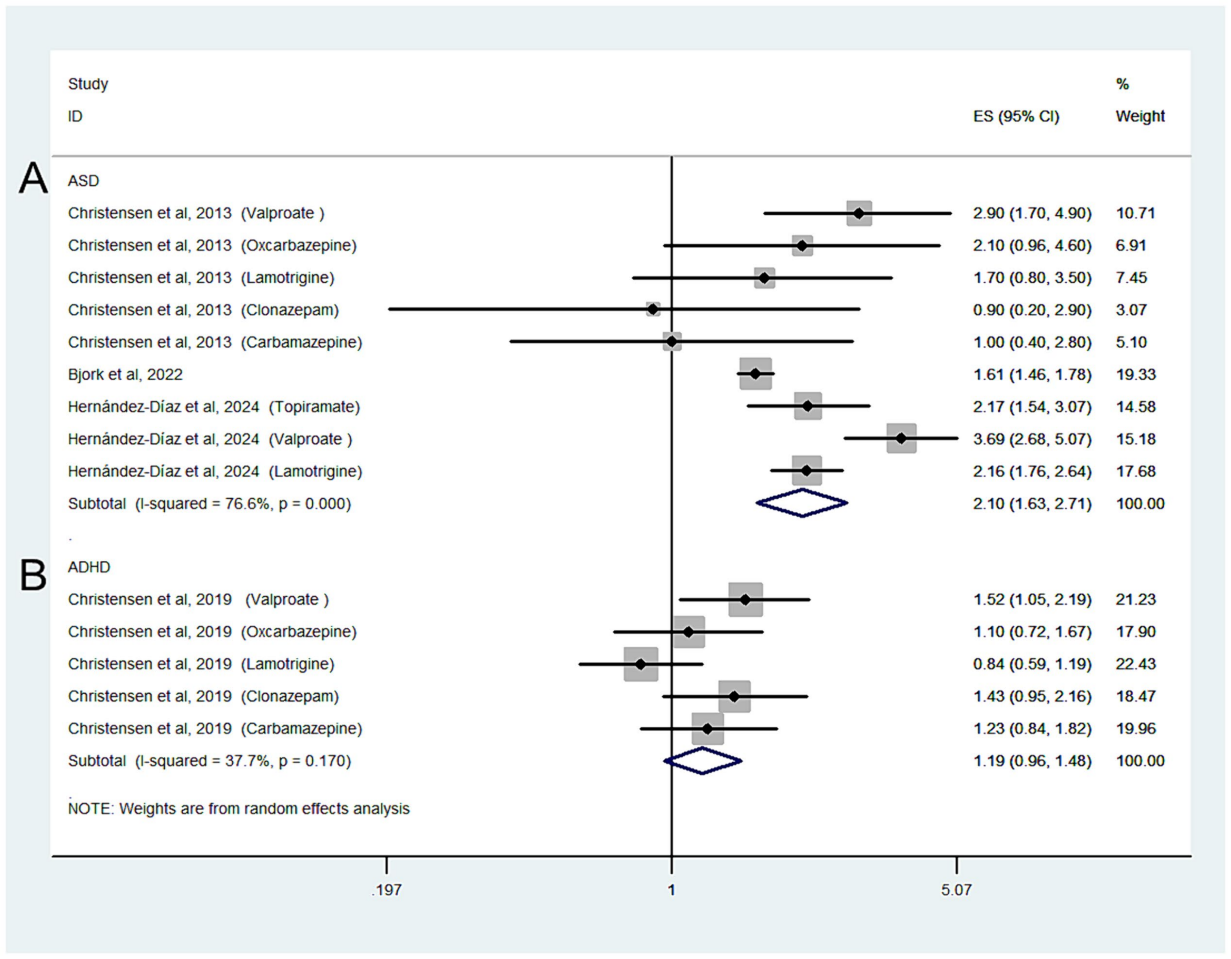
Forest plot of the overall risk of ASD **(A)** or ADHD **(B)** in relation to ASM exposure during pregnancy in general population.

Three studies with nine estimates reported the risk of ASD in relation to ASM exposure during pregnancy in the general population; the combined OR of the ASD risk was 2.1 (95% CI: 1.63–2.71; I^2^ = 76.6%, *p* < 0.001; Figure 2A).

When we grouped the studies by ASM type, significant associations were observed for mothers using valproate (OR: 2.29, 95% CI: 1.7–3.07; *p* < 0.001; I^2^ = 0%), topiramate (OR: 1.11, 95% CI: 0.84–1.47; *p* < 0.001; I^2^ = 0%), and oxcarbazepine (OR: 1.92, 95% CI: 1.37–2.69; *p* < 0.001; I^2^ = 0%), but not for those using lamotrigine (OR: 1.59, 95% CI: 0.95–2.6; *p* = 0.077; I^2^ = 89.2%), carbamazepine (OR: 1.32, 95% CI: 0.99–1.77; *p* = 0.062; I^2^ = 0%), or clonazepam (OR: 1.38, 95% CI: 0.94–2.01; *p* = 0.098; I^2^ = 0%).

One study with five estimates reported the risk of ADHD according to ASM exposure during pregnancy in the general population; the overall OR of ADHD was 1.19 (95% CI: 0.96–1.48; I^2^ = 37.7%, *p* = 0.118; Figure 2B).

#### Associations of ASM use with ASD and ADHD among mothers with a clinical indication

**Figure 3:**
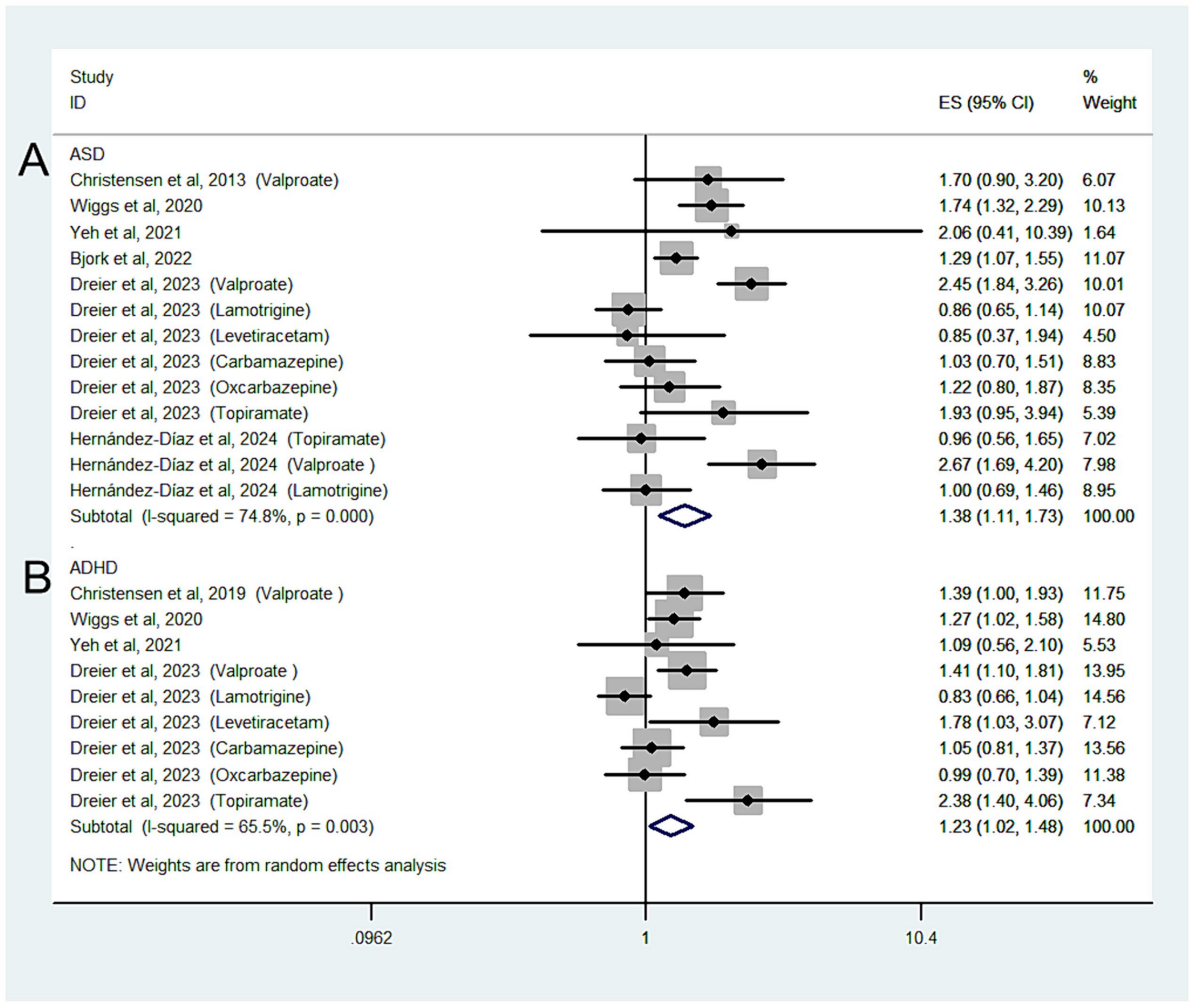
Forest plot of the overall risk of ASD **(A)** or ADHD **(B)** in relation to ASM exposure during pregnancy among mothers with a clinical indication.

Six studies with 13 estimates reported the risk of ASD according to ASM exposure during pregnancy in mothers with a clinical indication; the overall OR of ASD was 1.38 (95% CI: 1.11–1.73; I^2^ = 74.8%, *p* = 0.004; Figure 3A). We also performed a *post hoc* sensitivity analysis to evaluate the impact of including studies from the same database (Bjørk and Dreier et al.’s studies). Including only one study from the same database from the main analysis for the risk of ASD had no significant effect on the pooled OR.

When our analysis was limited to studies that only included mothers with epilepsy, the overall OR of ASD was 1.37 (95% CI: 1.1–1.73; *p* = 0.006; I^2^ = 76.8%).

When we grouped the studies by ASM type, significant associations were observed in mothers using valproate (OR: 2.38, 95% CI: 2.01–2.81; *p* < 0.001; I^2^ = 0%) but not in those using lamotrigine (OR: 0.87, 95% CI: 0.73–1.04; *p* = 0.123; I^2^ = 0%), carbamazepine (OR: 1.09, 95% CI: 0.87–1.37; *p* = 0.448; I^2^ = 0%), oxcarbazepine (OR: 1.27, 95% CI: 0.93–1.74; *p* = 0.14; I^2^ = 0%), levetiracetam (OR: 0.96, 95% CI: 0.54–1.68; *p* = 0.877; I^2^ = 0%), or topiramate (OR: 1.66, 95% CI: 0.87–3.18; *p* = 0.123; I^2^ = 66.2%).

Four studies with nine estimates reported the risk of ADHD in relation to ASM exposure during pregnancy in mothers with a clinical indication; the overall OR of the ADHD risk was 1.23 (95% CI: 1.02–1.48; I^2^ = 65.5%, *p* = 0.026).

When the analysis was limited to studies that only included mothers with epilepsy, the overall OR of ADHD was 1.25 (95% CI: 1.02–1.52; *p* = 0.029; I^2^ = 69.8%; Figure 3B).

When we grouped the studies by ASM type, significant associations were observed for mothers using valproate (OR: 1.49, 95% CI: 1.26–1.77; *p* < 0.001; I^2^ = 0%) but not for those using lamotrigine (OR: 0.87, 95% CI: 0.71–1.06; *p* = 0.166; I^2^ = 0%) or carbamazepine (OR: 1.11, 95% CI: 0.93–1.34; *p* = 0.246; I^2^ = 0%).

## Discussion

There has been increasing interest in the association between the use of ASMs during pregnancy and neurodevelopmental disorders in offspring. The findings of our study suggest that maternal exposure to ASMs during pregnancy was associated with a modestly increased risk of ASD or ADHD in offspring, even after controlling for potential confounders by indication. However, subgroup analyses based on individual ASMs found that only valproate was associated with an increased risk of ASD or ADHD, suggesting that the overall association may be driven by valproate use alone.

In our primary analyses, these associations were explored in the general population, and an almost two-fold increased risk of ASD was observed. ASMs are widely used to treat epilepsy and psychiatric disorders and have been associated with neurodevelopmental disorders in offspring (20, 21). Thus, enrolling healthy pregnant women unexposed to ASMs as a reference group may have overestimated this association. The ideal control for confounding by indication is a reference group of women with epilepsy or a psychiatric disorder who did not take an ASM during pregnancy. In our secondary analysis, these associations were explored among mothers with a clinical indication, and the risk of ASD was reduced 1.38-fold. When we further limited our analysis to pregnant mothers with epilepsy, the combined OR of ASD was 1.37. Although we reported inconsistent results for ADHD risk, these findings may have been limited by the small number of studies included in our analysis.

Animal studies have demonstrated different neurodevelopmental effects of prenatal individual ASM exposure (22). Several systematic reviews have also summarized the evidence regarding the neurobehavioral teratogenicity of intrauterine ASM exposure (23–25). Most clinical data are derived from case reports and small case series studies (26), and findings for different types of ASM have been inconsistent. Drawing conclusions from epidemiological studies has been hampered by differences in methodology and limited sample sizes. In primary analyses, three types of ASM were associated with an increased risk of ASD; however, these findings could not be replicated in a secondary analysis, in which only exposure to valproate during pregnancy was associated with an increased risk of ASD or ADHD in offspring. The precise biological mechanisms underlying the relationship are not entirely clear. Valproate is a short-chain low-molecular-weight fatty acid that quickly crosses the placental and blood–brain barriers (27). Valproate disrupts the metabolism of glutamate and neuronal plasticity in the fetal brain, thus triggering a neurodevelopmental disorder (28). In addition, valproate is a histone deacetylation histone deacetylase inhibitor, which affects gene replication and transcription (29). Results from a recent animal model study suggest that maternal exposure to valproate promotes replication and transcription of autism-associated genes, leading to autism-like behaviors (30). Finally, exposure to maternal valproate has been linked to congenital malformations (31), which are associated with an increased risk of neurodevelopmental disorders (32).

This meta-analysis is the first to provide an overall estimate of the effect of maternal ASM use during pregnancy on ASD and ADHD risk in offspring. The strength of our meta-analysis lies in the exclusive use of cohort studies, which are less prone to bias when assessing drug exposure during pregnancy. Second, the overall sample of all studies for the meta-analysis comprised >10 million pregnancy episodes across nine different countries, which makes the findings more generalizable and robust. Third, all of the studies were considered of high quality, which may minimize the bias of the review. Fourth, the associations were investigated in subgroup analyses based on ASM indication and type.

However, our review also had several limitations. The most important limitation of our meta-analysis was the significant heterogeneity among the studies, although in the subgroup analysis based on individual ASM, heterogeneity was reduced to 0%. Second, due to the inherent nature of observational studies, those included in this meta-analysis were prone to various types of confounding. We conducted analyses by pooling the adjusted data. Third, the etiology of epilepsy contributing to different ASM choice may affect the contributors toward ASD or ADHD, which may be an important confounding factor. Fourth, there were limited data in the studies on the dose or use of combination ASM agents; therefore, we could confirm that any exposure parameter was associated with neurodevelopmental outcome. Fifth, due to the small number of included studies, we could not evaluated the interactions between different ASM contribute to the incidence of autism or ADHD; however, the results of subgroup analyses based on individual ASM were consistent with the findings of two included studies (9, 12), which evaluated the risk of ADHD or ASD in the offspring of women who used antiepileptic drugs in monotherapy during pregnancy.

In conclusion, our results suggest that the link between prenatal ASM exposure and ASD or ADHD risk in children was attributable to the indication, rather than there being a causal relation. However, maternal use of valproate during pregnancy was associated with a significantly increased risk of ASD or ADHD, even compared to mothers with bipolar disorder or epilepsy who were not exposed to ASMs *in utero*.

## Data availability statement

The original contributions presented in the study are included in the article/Supplementary material, further inquiries can be directed to the corresponding author.

## Author contributions

S-CX: Writing – review & editing, Methodology, Conceptualization. YZ: Writing – review & editing, Resources, Project administration, Investigation. H-YJ: Writing – review & editing, Visualization, Investigation, Formal analysis. JT: Writing – review & editing, Writing – original draft, Validation, Supervision, Funding acquisition, Formal analysis.
