# Supplementary material for: Exposure to anti-seizure medication during pregnancy and the risk of autism and ADHD in offspring: a systematic review and meta-analysis
Source: Front Neurol. 2024 Jul 22;15:1440145. doi: 10.3389/fneur.2024.1440145 (PMC11298387; doi:10.3389/fneur.2024.1440145)
Supplement: Supplementary file 1 [file Table_1.docx]

| **Table S1** NOS for Assessment of Quality of Included Studies: Cohort Studies | | | | | |  |  |  |
| --- | --- | --- | --- | --- | --- | --- | --- | --- |
| Study | Selection |  |  |  | Comparability |  | Outcomes |  |
|  | Representativeness of exposed cohort? | Selection of the nonexposed cohort? | Ascertainment of exposure? | Demonstration that outcome of interest was not represent at the start of the study | Comparability of Cohort* | Assessment of outcome | Was follow-up long enough for outcomes to occur | Adequacy of follow up of cohorts |
| Christensen et al, 2013 | ★ | ★ | ★ | ★ | ★★ | ★ | ★ | ★ |
| Christensen et al, 2019 | ★ | ★ | ★ | ★ | ★★ | ★ | ★ | ★ |
| Wiggs et al, 2020 | ★ | ★ | ★ | ★ | ★★ | ★ | ★ | ★ |
| Yeh et al, 2021 | ★ | ★ | ★ | ★ | — | ★ | ★ | ★ |
| Bjork et al, 2022 | ★ | ★ | ★ | ★ | ★★ | ★ | ★ | ★ |
| Dreier et al, 2023 | ★ | ★ | ★ | ★ | ★★ | ★ | ★ | ★ |
| Hernández-Díaz et al, 2024 | ★ | ★ | ★ | ★ | ★★ | ★ | ★ | ★ |
| Note: A star denotes a score of 1; * A maximum of 2 stars can be allotted in this category | | | | | | | |  |
